# Commentary: AI-based online chat and the future of oncology care: a promising technology or a solution in search of a problem?

**DOI:** 10.3389/fonc.2023.1239932

**Published:** 2023-09-07

**Authors:** Hui Zhang, Yongfu Guan, Jinping Chen, Wenting Tong

**Affiliations:** ^1^ Department of Rehabilitation and Elderly Care, Gannan Healthcare Vocational College, Ganzhou, China; ^2^ Department of Pharmacy, Gannan Healthcare Vocational College, Ganzhou, China

**Keywords:** global healthcare resource allocation, data privacy, language bias, legal and ethical challenges, ChatGPT

Joseph Kassab’s recent article (1) inspired us to consider the potential impact of AI chatbots like ChatGPT on the global healthcare and the challenges that must be addressed to harness their full potential. One of the key challenges is the severe imbalance in the distribution of global healthcare resources, particularly in some regions of Asia, Africa, and Latin America. The causes of these disparities include historical legacies, cultural backgrounds, political systems, technological infrastructure, and economic development. Various international organizations, charities, and governmental and non-governmental organizations (NGOs) have implemented measures such as financial assistance, technical support, and human resource training to address this issue, but achieving equitable distribution of global healthcare resources still requires increased investment and collaboration (2).

ChatGPT is a powerful language model developed by OpenAI based on the GPT-3/GPT-4 architecture that has demonstrated remarkable accuracy and broad potential for application in medical diagnosis. Although some research suggests that ChatGPT needs to be specifically trained in the medical field to be effective (3). a substantial body of research has already demonstrated the excellent capabilities of ChatGPT in the medical field (4, 5).

Collaboration with medical experts is essential to regularly assess and validate its diagnostic suggestions to ensure accuracy and reliability. Additionally, since ChatGPT cannot provide medical diagnoses or treatment plans autonomously, such systems cannot be licensed for medical practice.

Another challenge is privacy concerns. Disputes over privacy data on a global scale have led to the prohibition of ChatGPT usage in some countries, countries such as Russia, Italy, and China have either banned or previously imposed bans on the use of ChatGPT. To overcome an issue that may be more political than scientific or privacy-related, stricter data protection policies and regulations could be established to ensure data is utilized in compliance with laws while safeguarding user privacy.

Moreover, limited access to patient data and language bias are other challenges that need to be addressed. Also, due to limitations in training data, ChatGPT exhibits a significant performance gap between English and non-English scenarios, preventing non-English speakers from fully benefiting from its resources. To resolve these issues, the collection and training of non-English data should be expanded to enhance AI performance across various language environments.

Furthermore, OpenAI has restricted access to ChatGPT for users in some countries, leading to regional bias. Collaboration with relevant countries and regions should be pursued to jointly explore potential technology applications and proactively expand the scope of services, ensuring that users worldwide can benefit. Fee issues also need to be addressed, as the fee-based model may lead to accessibility imbalances among countries with different income levels. Flexible pricing strategies can be adopted, offering various pricing schemes based on national income levels and user needs, ensuring that everyone can enjoy the conveniences brought by AI.

Finally, legal liability and sensitivity to problem nuances are other challenges that need to be considered. Further refinement of the legal system is needed to clarify the legal responsibility for AI decision-making. Subtle differences between questions may yield different answers, and continuous optimization of AI algorithms is required to improve the understanding and accuracy of responses.

Although AI technologies like ChatGPT face numerous challenges, addressing these issues requires a multifaceted approach, incorporating technological innovation, ethics, law, data security, and education. The advancement of AI is inevitable, and it is essential to adopt a proactive and flexible mindset towards tackling these challenges. Governments, businesses, research institutions, and the public should collaborate, share resources and knowledge, and expand cooperation to optimize AI technology and improve AI applications in healthcare. Additionally, it is important to address other elements influencing healthcare resource distribution, such as infrastructure development, medical personnel training, and public health policies. AI technology can complement and enhance traditional healthcare systems, but it cannot replace them entirely. Focusing on resolving issues within conventional healthcare systems is also crucial to deliver more comprehensive, efficient, and sustainable medical services to the public.

## Author contributions

HZ led the conceptualization of the study, literature review, and initial drafting. YG provided insights into AI technology in healthcare, including challenges and potential solutions, and contributed to manuscript revision. JC examined privacy and data security issues, also contributing to manuscript development. WT explored socio-political implications and discussed disparities in AI accessibility, participating in manuscript writing and editing. All authors approved the final version and agreed to be accountable for their own contributions.
