# Corrigendum: Commentary: AI-based online chat and the future of oncology care: a promising technology or a solution in search of a problem?

**DOI:** 10.3389/fonc.2023.1334176

**Published:** 2023-12-07

**Authors:** Hui Zhang, Yongfu Guan, Jinping Chen, Wenting Tong

**Affiliations:** ^1^ Department of Rehabilitation and Elderly Care, Gannan Healthcare Vocational College, Ganzhou, China; ^2^ Department of Pharmacy, Gannan Healthcare Vocational College, Ganzhou, China

**Keywords:** global healthcare resource allocation, data privacy, language bias, legal and ethical challenges, ChatGPT

**Incorrect Affiliation**

In the published article, there was an error in affiliation(s) 1 and 2. The published affiliations stated: “1 Department of Rehabilitation and Elderly Care, Gannan Health Vocational College, Ganzhou, China; 2 Department of Pharmacy, Gannan Health Vocational College, Ganzhou, China”, the corrected affiliations are: “1 Department of Rehabilitation and Elderly Care, Gannan Healthcare Vocational College, Ganzhou, China; 2 Department of Pharmacy, Gannan Healthcare Vocational College, Ganzhou, China”.

The authors apologize for this error and state that this does not change the scientific conclusions of the article in any way. The original article has been updated.

